# Ecology, feeding and natural infection by *Leishmania* spp. of phlebotomine sand flies in an area of high incidence of American tegumentary leishmaniasis in the municipality of Rio Branco, Acre, Brazil

**DOI:** 10.1186/s13071-018-2641-y

**Published:** 2018-01-26

**Authors:** Márcia Moreira de Ávila, Andreia Fernandes Brilhante, Cristian Ferreira de Souza, Paula Dias Bevilacqua, Eunice Aparecida Bianchi Galati, Reginaldo Peçanha Brazil

**Affiliations:** 1 Federal Institute of Acre, Rio Branco, Acre Brazil; 2 Faculty of Public Health, University of São Paulo, São Paulo, Brazil; 3 Oswaldo Cruz Institute, IOC/FIOCRUZ, Rio de Janeiro, Brazil; 4 Federal University of Viçosa, Minas Gerais, Brazil

**Keywords:** Tegumentary leishmaniasis, Diversity, Ecology, Vectors, *Leishmania* spp.

## Abstract

**Background:**

Phlebotomine sand flies (Diptera: Psychodidae) are insects of medical importance due to their involvement in the zoonotic transmission of *Leishmania* spp. to vertebrates. The aim of this work was to study the ecology of the sand fly fauna of two types of environments, a rural environment (the Transacreana Road) and an urban park (Horto Florestal Park), both located in the municipality of Rio Branco in the state of Acre, Brazil. Additionally, this study intended to investigate *Leishmania* infection and blood meal sources of these sand flies using molecular techniques.

**Methods:**

The sand fly fauna was studied in different environments (i.e. forest and peridomestic environments in a rural area, and an urban forest) using Shannon traps and HP light traps to collect sand fly specimens over 13 consecutive months (December 2014 to January 2016). For investigating natural infection by *Leishmania* and the source of sand fly blood meals, DNA samples were extracted from female sand flies and subjected to polymerase chain reaction targeting ITS1 and *cytb* genes. DNA sequencing was subsequently used to identify species of *Leishmania* and the source of blood meals.

**Results:**

A total of 2515 individual sand flies of 43 species were collected and identified*, Trichophoromyia auraensis* (839; 33.35%), *Trichophoromyia* spp. (537; 21.35%) and *Evandromyia saulensis* (187; 7.43%) were more abundant in the rural area (S = 41 species) than in the urban forest. No significant differences were found in species richness between forest and peridomestic environments in the rural area (*H* = 0.04; *P* > 0.05), but a larger number of species was found in the forest. *Leishmania* DNA was sequenced in 13 samples, confirming the presence of *L*. (*V*.) *braziliensis* in *Th*. *auraensis* (*n* = 1), *Ev*. *saulensis* (*n* = 2), *Ev*. *walkeri* (*n* = 1), *Ps*. *llanosmartinsi* (*n* = 1), *Pi*. *nevesi* (*n* = 2), *Ps*. *davisi* (*n* = 1), *Ps*. *ayrozai* (*n* = 1), *Pa*. *aragaoi* (*n* = 1), *Ny*. *antunesi* (*n* = 1) and *Ev*. *infraspinosa* (*n* = 1). Only *Ps*. *ayrozai* possessed a sequence similar to that of *L*. (*V*.) *guyanensis* (99%). Through microscopic analysis, five specimens of *Ev. saulensis* were found to possess flagellate forms in the hindgut, with an infection rate of 2.4%. Samples from 33 fed females were submitted to *cytb* gene amplification, for which sequencing determined that all were similar to the sequence deposited on GenBank for *Gallus gallus* (domestic chicken).

**Conclusions:**

The high abundance of *Trichophoromyia auraensis* and *Ev. saulensis*, and the detection of *L*. (*V*.) *braziliensis* DNA, suggests that both species may be vectors of American tegumentary leishmaniasis. *Psychodopygus ayrozai* was found to be infected by *L*. (*V*) *braziliesnsis* and *L*. (*V*.) *guyanensis*, and although collected in low abundance, it may be a potential vector in the region. The sand fly fauna was found to be rich and diverse with predominance of the genus *Psychodopygus*. Identification of food sources of fed females showed that 100% amplified a gene region compatible with the domestic chicken, which although considered refractory in the disease transmission cycle, may have an influence on the population dynamics of sand flies.

## Background

Leishmaniasis is a complex of vector-transmitted zoonotic diseases, of great clinical importance and epidemiological diversity. It is caused by protozoan parasites of the genus *Leishmania* (order Kinetoplastida, family Trypanosomatidae) in vertebrate hosts including man. Transmission is performed by hematophagous insects of the subfamily Phlebotominae (family Psychodidae), known as sand flies, which have complex ecological behavior [1–3]. There are two main clinical types of leishmaniasis, which are caused by different protozoan species of the genus *Leishmania*: visceral leishmananiasis (VL) in which the etiological agent is *Leishmania infantum* and tegumentary leishmaniasis (TL) that affect the skin and mucous membranes [1, 4]. American tegumentary leishmaniasis (ATL) is a significant disease in Brazil due in part to the great abundance of sand fly vectors of the various species of *Leishmania* that occur in the country [3–6]. With 20,187 cases reported in 2015 [7], ATL has been reported in all states, suggesting adaptation of the parasites and their vectors to anthropic environments [8–11]. They have adapted to urban environments that are surrounded by to forest remnants, also because of the greater supply of food sources for the vectors in the form of domestic animals in peridomestic areas [11, 12]. In the North Region of Brazil, ATL is endemic with 45% (9278) of the confirmed cases for the country in 2015 [7]. This region is characterized by human occupation associated with agricultural activities, mineral extraction and intense deforestation. It is also a region possessing low human development indices, with the absence of basic services such as sanitation in most residences, which are considered potential sites for the occurrence of tropical diseases [12–14].

In the state of Acre, located southwest of the Amazon in Brazil, ATL is considered endemic with high rates of incidence and prevalence [11, 15, 16]. Of the few studies that have been undertaken in the region, there have been reports of *Leishmania* (*Viannia*) *guyanensis*, *Leishmania* (*V*.) *braziliensis*, *Leishmania* (*V*.) *lainsoni* and *Leishmania* (*Leishmania*) *amazonensis* [17–19]. Likewise, these studies have reported the sand fly species *Bichromomyia flaviscutellata*, *Migonemyia migonei*, *Nyssomyia whitmani*, *Ny. antunesi*, *Ny. umbratilis*, *Psychodopygus ayrozai*, *Ps*. *davisi*, *Ps*. *paraensis* and *Trichophoromyia ubiquitalis* as the main vectors of the etiological agents of leishmaniasis [11, 16, 20–22]. Rio Branco is a municipality in Acre with the greatest occurrence of the disease, with 500 ATL cases reported between 2013 and 2014 [7]. However, studies of leishmaniasis are still incipient, especially with regard to sand fly ecology and food sources, and the transmission cycle of *Leishmania* spp. In addition, little information has been acquired regarding natural infection of possible vectors by dissection and observation of trypanosomatids [23–25], or by molecular methods such as polymerase chain reaction (PCR), which are capable of identifying *Leishmania* DNA, such the studies carried out in Assis Brasil [22] and Rio Branco [26].

The objective of this study was to investigate the sand fly fauna of rural and urban environments, and determine their food source and natural infection by *Leishmania* in an endemic area of ATL in Rio Branco.

## Methods

### Study area

**Fig. 1:**
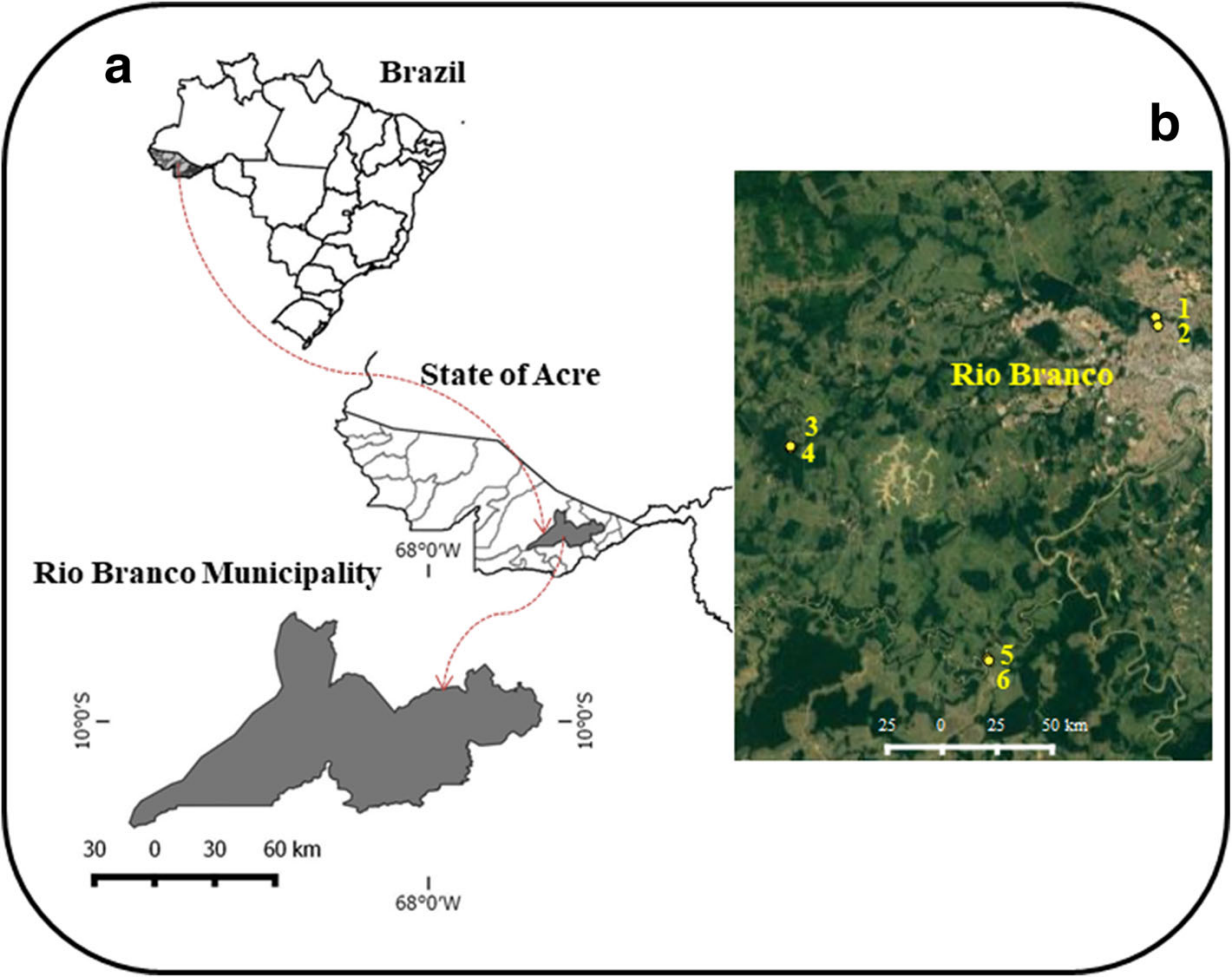
Map of the study area with (**a**) location of the collection area in the municipality of Rio Branco, and (**b**) sand fly sampling areas in the urban park (1 and 2, Horto Florestal park) and rural (3 and 5, Dom Joaquim’s settlement, 5 and 6, Riozinho Branch), environments, municipality of Rio Branco Acre, Brazil (Map data ©2017 Google)

The municipality of Rio Branco (09°59′11"S, 67°49′52"W), capital of the state of Acre (AC), is located in the southwestern part of the Amazon Region of northern Brazil (Fig. 1a). It encompasses an area of 8835.52 km^2^ [27], and possesses an estimated population of 377,057 inhabitants [27]. The vegetation is composed of dense and open tropical forest with predominance of bamboo and palm trees. The climate is equatorial, with temperatures varying from 24 °C to 32 °C and annual rainfall of between 1877 and 1982 mm. The hottest period of the year corresponds to the months of July to August, marked by intense fires due to the conversion of forest to pastures [28].

### Sand fly sampling

Sand flies were collected in forest and peridomestic environments in a rural area, and in an urban forest, between December 2014 and January 2016. Sampling was carried out once a month using six HP light traps, set at 6:00 pm and retrieved at 6:00 am, distributed among the following sampling sites: Dom Joaquim Settlement Pole - Highway AC-90 (9°59′46.6"S, 67°58′51.0"W); Ramal do Riozinho - Highway AC-90 (10°05′00.1"S, 67°53′56.0"W); and Horto Florestal Urban Environmental Park (9°56′39.0"S, 67°49′43.6"W) (Fig. 1b).

To determine sand fly anthropophily, nocturnal sampling (06:00 pm to 08:00 pm) was performed by 2 persons using a Shannon trap in an area of peridomiciliar forest at Dom Joaquim Settlement during 13 nights. The females collected during this sampling were killed with ethyl acetate and dissected on slides with a drop of sterile saline solution to expose and examine the digestive tract and spermathecae for flagellate research and species identification, respectively. The specimens were then stored, in microtubes containing absolute ethanol, in pools of up to 10 females according to the month of collection and species, for subsequent molecular detection of *Leishmania* spp.

Fed females collected in HP light traps had only their genitalia exposed for species identification, and then were stored in 90% absolute ethanol for food source analysis.

All the remaining specimens collected in the Shannon trap were clarified according to Forattini [29] and identified according to the taxonomic key proposed by Galati [30]. The abbreviation of species names followed Marcondes [31].

### DNA extraction and PCR conditions

After being identified, female sand flies were subjected to DNA extraction using the Gentra Puregene® Cell and Tissue Extraction Kit (QIAGEN, Hilden, Germany), following the manufacturer’s protocol.

After DNA extraction, PCR was performed targeting the internal transcribed spacer 1 (ITS1) using the primers LITSR - Forward (5′-CTG GAT CAT TTT CCG ATG-3′) and L5.8S - Reverse (5′-TGA TAC CAC TTA TCG CAC TT-3′) [32], to detect natural infection by *Leishmania* spp. In non-engorged females, the region amplifies a 350 bp fragment by means of the following reaction: 1× buffer solution (200 mM Tris-HCl, pH 8.4; 500 mM KCl), 1.5 mM MgCl_2_, 0.2 mM dNTPs, 0.5 pmol of the LITSR primer, 0.5 pmol of the L5.8S primer, 1 U of Taq DNA Polymerase Platinum (Invitrogen, California, USA) of 5 μl template DNA in a total volume of 25 μl. Samples were amplified in an automatic thermocycler (MaxyGene Gradient - AXYGEN, Corning, USA) with 33 cycles of denaturation at 95 °C for 30 s, annealing at 53 °C for 1 min and extension at 72 °C for 1 min. In negative control, pure water was used with DNA from male sand flies. The amplified product was analyzed on a 2% agarose gel stained with GelRed (Nucleic Acid Gel Stain - Biotium, Fremont, USA) and compared with amplified material from positive samples from reference strains of *Leishmania braziliensis* (MHOM/BR/75/M2903). Positive samples were cloned into competent *Escherichia coli* (DH5-α) cells using the CloneJet PCR cloning kit (Thermo Scientific, California, USA) following the manufacturer’s protocol. The colonies of transformed bacteria were selected, re-submitted to PCR of the ITS1 gene and sequenced.

### Blood-feeding study and PCR conditions

DNA extracted from blood-fed female sand flies was submitted to PCR for amplification of the Cytochrome B gene, which amplifies a fragment of approximately 359 bp using the *cytb* primers (forward: 5′-CCA TCC AAC ATC TCA GCA TGA TGA AA-3′; and reverse: 5′-GCC CCT CAG AAT GAT ATT TGT CCT CA-3′) [33]. DNA extraction from the females followed the same protocol as described previously but in a laminar flow booth and adopting disinfection measures with care not to contaminate samples with human DNA. The PCR reaction was performed using 1× buffer solution (200 mM Tris-HCl, pH 8.4; 500 mM KCl), 1.5 mM MgCl_2_, 0.2 mM dNTPs, 0.5 pmol LITSR primer, 0.5 pmol L5.8S primer, 1 U of Taq DNA polymerase platinum (Invitrogen, California, USA) and 5 μl template DNA, in a total volume of 50 μl. Samples were amplified in an automatic thermocycler (MaxyGene Gradient - AXYGEN, Corning, USA) with 32 cycles of 95 °C for 20 s (denaturation), 53 °C for 30 s (annealing) and 72 °C for 1 min (extension). As negative control, pure autoclaved water and DNA of male and female sand flies without the presence of intestinal blood were used. Five μl of the amplified product was analyzed on a 2% agarose gel stained with 0.1 μl of GelRed (Biotium Acid Gel Stain - Biotium, Fremont, USA) and compared to the band obtained by positive control (DNA amplified from canine clot).

### Sequencing

For both the study of natural infection and that of food source, amplified PCR products were sent to Macrogen (South Korea) for sequencing using a 3730XL Applied Biosystems. The sequences obtained were edited using the program Sequencher, compared by BLAST with sequences available in GenBank.

### Data analysis

Kruskal-Wallis was used to test the hypothesis of equality among the ecological descriptors in the different ecotopes studied. The measures of diversity among the environments were obtained using the Shannon-Wiener’s (*H′*) diversity index, the Pielou’s equitability index (*J’*), Margalef’s richness index and the Berger-Parker’s dominance index [34], followed by comparisons and Spearman’s correlation tests between species and climatic variables (temperature, relative humidity and rainfall). The analyses were performed using the Past 2.17c program and IBM SPSS 20 software, with a 5% level of significance. The minimum natural infection rate was calculated considering the number of positive pools multiplied by 100 and divided by the number of insects tested [35].

## Results

A total of 2515 sand flies were collected of 43 species belonging to 13 genera: *Brumptomyia*, *Bichromomyia*, *Evandromyia*, *Lutzomyia*, *Micropygomia*, *Migonemyia*, *Nyssomyia*, *Pintomyia*, *Pressatia*, *Psathiromyia*, *Psychodopygus*, *Sciopemyia* and *Tricophoromyia* (Table 1). The genus *Psychodopygus* was the most frequently collected (17.0% of the total), followed by *Evandromyia* (10.0%), *Thricophoromyia* (10.0%) and *Brumptomyia* (10.0%). *Evandromia saulensis* was the third most abundant species in the collections (7.4%), and occurred in all of the studied ecotopes, but predominantly in the rural area (Table 2).

**Table 1:** Abundance of sand flies collected (with CDC and Shannon traps), during the study period of December 2014 to January 2016 in Rio Branco, Acre, Brazil

Environment/Species	Rural area	Urban area	Total
	AC-90 road	Urban park	
	(*n*)	(*n*)	
*Bi. flaviscutellata*	51	6	57
*Br. avellari*	9	0	9
*Br. brumpti*	0	1	1
*Br. pentacantha*	2	1	3
*Brumptomyia* sp.	7	1	8
*Ev. infraspinosa*	2	1	3
*Ev. andersoni*	–	1	1
*Ev. saulensis*	174	13	187
*Ev. walkeri*	85	3	88
*Lu. evangelistai*	1	0	1
*Lu. gomezi*	1	0	1
*Lu. sherlocki*	17	4	21
*Mi. micropyga*	1	3	4
*Mi. trinidadensis*	3	–	3
*Micropygomia* (*Sauromyia*) sp.	2	0	2
*Mg. migonei*	2	1	3
*Ny. antunesi*	35	23	58
*Ny. shawi*	7	0	7
*Ny. whitmani*	22	17	39
*Pa. abonnenci*	1	0	1
*Pa. aragoia*	1	0	1
*Pa. abunaensis*	1	0	1
*Pa. bigeniculata*	4	0	4
*Psathyromyia* sp.	2	0	2
*Pi. nevesi*	61	14	75
*Pi. serrana*	8	2	10
*Pr. calcarata*	132	6	138
*Pr. choti*	42	3	45
*Pressatia* sp.	84	2	86
*Ps. carrerai carrerai*	97	2	99
*Ps. llanosmartinsi*	7	–	7
*Ps. amazonensis*	1	–	1
*Ps. ayrozai*	5	–	5
*Ps. claustrei*	7	–	7
*Ps. davisi*	89	1	90
*Ps. hirsutus hirsutus*	47	–	47
*Sc. servulolimai*	1	1	2
*Sciopemyia* sp.	7	4	11
*Th. auraensis*	774	65	839
*Th. brachipyga*	11	1	12
*Trichophoromyia* sp.	500	37	537
*Th .ubiquitalis*	1	0	1
Total	2304*	213*	2517

*Comparison for the abundance of collected sand flies in the rural and urban environment (Kruskal-Wallis test*, H* = 17,4, *df* = 42, *P* = 0.0002)

**Table 2:** Abundance and diversity indices for sand flies collected from December 2014 to January 2016 in Rio Branco, Acre, Brazil

	Area I(Rural)			Area II (Urban)		Total	%
Environment	Extradomiciliar		Peridomiciliar	Wild			
Ecotope	Forest/ CDC trap	Forest/ Shannon trap	Chicken/ CDC trap	Forest 1	Forest 2		
Species	*n*	*n*	*n*	*n*	*n*		
*Bi. flaviscutellata*	42	5	4	3	3	57	2.26
*Br .avellari*	1	–	8	–	–	9	0.36
*Br. brumpti*	–	–	–	–	1	1	0.04
*Br. pentacantha*	–	–	2	1	–	3	0.12
*Brumptomyia* sp.	2	3	2	–	1	8	0.32
*Ev. infraspinosa*	1	1	–	–	1	3	0.12
*Ev. andersoni*	–	–	–	1	–	1	0.04
*Ev. saulensis*	81	66	27	10	3	187	7.43
*Ev. walkeri*	30	14	41	2	1	88	3.50
*Lu. evangelistai*	1	–	–	–	–	1	0.04
*Lu. gomezi*	1	–	–	–	–	1	0.04
*Lu. sherlocki*	6	7	4	2	2	21	0.83
*Mi. micropyga*	–	–	1	2	1	4	0.16
*Mi. trinidadensis*	1	–	2	–	–	3	0.12
*Micropygomia* (*Sauromyia*) sp*.*	1	–	1	–	–	2	0.08
*Mg. migonei*	1	–	1	–	1	3	0.12
*Ny. antunesi*	11	8	16	21	2	58	2.30
*Ny. shawi*	4	–	3	–	–	7	0.28
*Ny. whitmani*	11	9	2	10	7	39	1.55
*Pa. abonnenci*	–	1	–	–	–	1	0.04
*Pa. aragoia*	–	1	–	–	–	1	0.04
*Pa. abunaensis*	1	–	–	–	–	1	0.04
*Pa. bigeniculata*	4	–	–	–	–	4	0.16
*Psathyromyia* sp.	2	–	–	–	–	2	0.08
*Pi. nevesi*	41	13	7	11	3	75	2.98
*Pi. serrana*	–	–	8	2	–	10	0.40
*Pr. calcarata*	32	2	98	6	–	138	5.48
*Pr. choti*	3	4	35	2	1	45	1.79
*Pressatia* sp.	16	5	63	2	–	86	3.42
*Ps. carrerai carrerai*	55	29	13	–	2	99	3.93
*Ps. llanosmartinsi*	–	7	–	–	–	7	0.28
*Ps. amazonensis*	–	1	–	–	–	1	0.04
*Ps. ayrozai*	–	5	–	–	–	5	0.20
*Ps. claustrei*	1	6	–	–	–	7	0.28
*Ps. davisi*	17	48	24	–	1	90	3.58
*Ps. hirsutus hirsutus*	34	8	5	–	–	47	1.87
*Sc. servulolimai*	1	–	–	–	1	2	0.08
*Sciopemyia* sp.	4	1	2	–	4	11	0.44
*Tr. auraensis*	482	22	270	13	52	839	33.33
*Tr. brachipyga*	8	–	3	1	–	12	0.48
*Trichophoromyia* sp.	275	12	213	3	34	537	21.33
*Tr. ubiquitalis*	–	1		–	–	1	0.04
Total (*n*)	1170	279	855	92	121	2517	100.0
Diversity indices							
Richness (S)	32^a^	–	26^a^	17	20		
Shannon-Wiener’s (H′)	1.99^b^	–	2.11^b^	2.39^b^	1.85^b^		
Pielou’s (J’)	0.57	–	0.64	0.84	0.61		
Berger-Parker’s (D)	0.41	–	0.31	0.22	0.42		

^a^Comparison for species richness (S) of sand flies collected in peri- and extra-domiciliary areas (Kruskal-Wallis test*, H* = 0,04, *df* = 1, *P* = 0.0531)

^b^Comparison for the diversity index (H′) between Area I and Area II (Kruskal-Wallis test, *H* = 1, *df* = 1, *P* = 0.317)

**Fig. 2:**
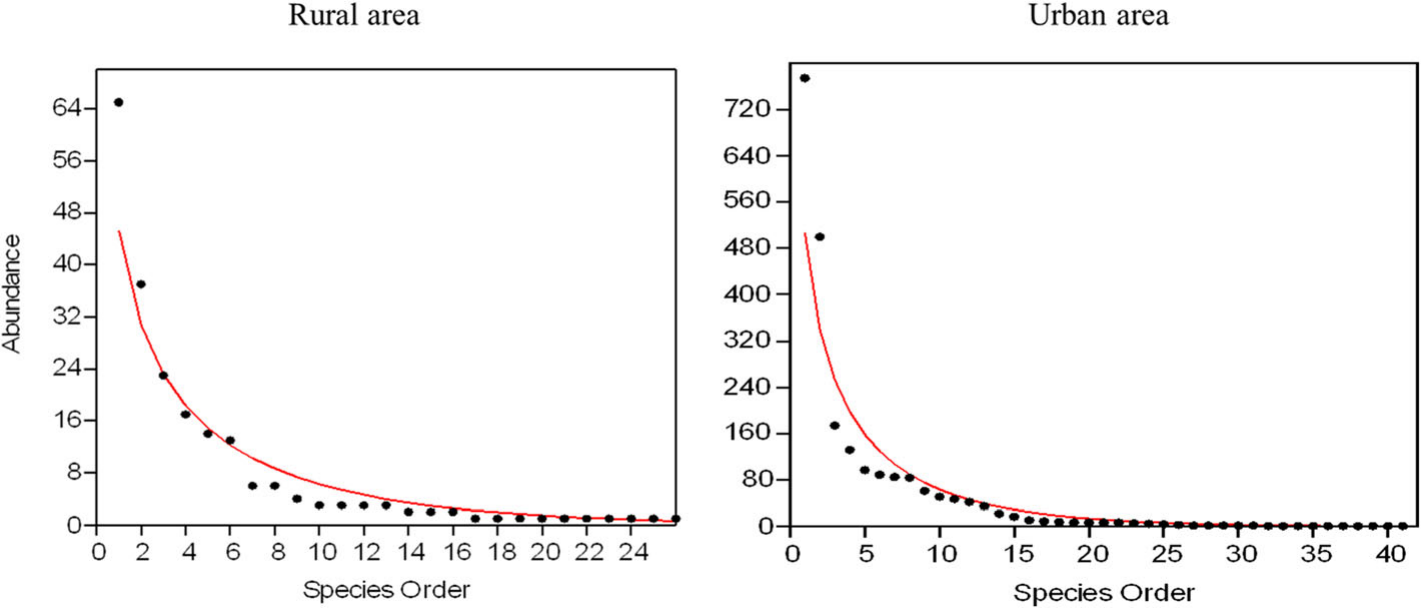
Abundance curves of sand fly species collected in the rural and urban park environments, from December 2014 to January 2016, in the municipality of Rio Branco Acre, Brazil

*Evandromia saulensis* (*n* = 66) was the most frequently captured species in Shannon traps, indicating the anthropophilic behavior of this species, followed by *Psychodopygus davisi* (*n* = 48). A total of 2304 specimens of 41 species were collected at rural sites, with the most abundant being *Th*. *auraensis*, *Trichophoromyia* sp., *Ev.saulensis*, *Pr*. *calcarata* and *Ps*. *carrerai carrerai.* In the urban park, 213 specimens of 26 species were collected, with the most abundant being *Th*. *auraensis, Trichophoromyia* sp. and *Ny*. *antunesi.* Sand fly abundance was higher in the forest and peridomestic environments in the rural area than in the urban forest (*H* = 17.9, *df* = 42, *P* < 0.05) (Fig. 2). In the rural area, mean species richness was 5.16 (*S* = 41) and mean equitability (*J’*) 0.61, whereas in the urban park the mean richness was lower at 4.66 (*S* = 26), and equitability higher (*J’* = 0.72), since it is an environment with a more uniform pattern of abundance. The diversity index was higher in the urban forest, but not significantly different from that of the rural area according to the Kruskal-Wallis test (*H* = 1, *df* = 1, *P* > 0.05) for two samples. Species dominance, according to the Berger-Parker’s index, averaged 0.33 and 0.30 for the rural area and urban forest, respectively. There was a significant correlation between the total number of sand flies collected and mean temperature on the days of collection (Spearman’s rho = 0.58, *n* = 13, *P* = 0.03) (Table 3); however, there was no significant correlation between the number of sand flies collected by HP light traps and relative humidity (Spearman’s rho = -0.66, *n* = 13, *P* = 0.831) or monthly rainfall (Spearman’s rho = -0.38, *n* = 13, *P* = 0.45) during the study period. Species richness for forest and peridomestic environments in the rural area did not differ significantly (*H* = 0.04, *df* = 1, *P* > 0.05), although there was a greater number of species in the forest. The greatest diversity (Shannon index, H′) of species was in the primary forest of the urban forest, with 17 species (H′ = 2.39), followed by rural peridomestic environments (H′ = 2.11) with 26 species, rural forest (H′ = 1.99) with 32 species and secondary forest in the urban forest (H′ = 1.85) with 20 species (Table 2).

**Table 3:** Abundance of sand flies collected in CDC light traps and monthly means for temperature, relative humidity and rainfall from December 2014 to January 2016 in Rio Branco, Acre, Brazil

Collection period	Total no. of sand files collected	Percent of total	Temperature (°C)	Humidity (%)	Rainfall (mm)
December 2014	603	26.9	32.2	76.6	149.2
February 2015	102	4.6	32.1	70.4	292.6
May 2015	116	5.2	30.9	76.3	351.3
April 2015	110	4.9	29.7	75.4	231.7
May 2015	51	2.3	31.0	74.4	256.3
June 2015	31	1.4	29.2	65.3	31.0
July 2015	128	5.7	29.3	60.7	9.5
August 2015	26	1.2	28.3	64.7	45.2
September 2015	158	7.1	35.6	51.7	80.7
October 2015	248	11.1	32.3	57.3	96.0
November 2015	100	4.5	33.4	60.2	292.4
December 2015	412	18.4	32.6	65.9	171.8
January 2016	153	6.8	33.4	59.0	132.6
Total	2238^a^	100	31.5^b^	66.0^a^	2140.3^a^

*Note*: Equal letters indicate a non-significant correlation between the total number of sand flies collected and temperature, relative humidity and rainfall (*P* > 0.05); and different letters indicate a significant correlation (*P* < 0.05)

### *Leishmania* infection in sand flies

**Fig. 3:**
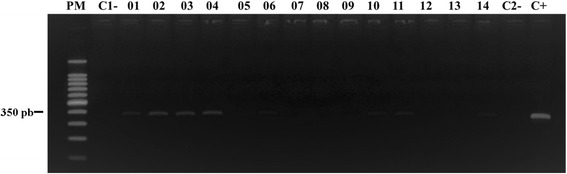
PCR-ITS1-2% agarose gel, colored with GelRed showing ITS1 PCR amplification product from DNA extracted from female sand flies collected in the municipality of Rio Branco, Acre, Brazil. Lanes 1–4, 6, 10, 11 and 14: collected female sand flies positive for natural infection with *Leishmania* sp.; Lanes C1- and C2-: negative controls (Milli-Q ultrapure water); Lane C+: positive control (*Leishmania* (*Viannia*) *braziliensis* DNA) (MHOM/BR/75/M2903)

A total of 206 female sand flies were collected using Shannon traps in a forest environment and used to determine natural infection by microscopy and molecular analysis. Sand fly species were grouped into 39 pools and 43 individual samples. Of these, 17 pools (*Th*. *auraensi*, 3 pools; *Ev*. *saulensis*, 2; *Ev*. *walkeri*, 2; *Bi*. *flaviscutellata*, 1; *Ps*. *llanosmartinsi*, 1; *Pi*. *nevesi*, 3; *Ps*. *davisi*, 2; *Ps*. *ayrozai*, 2; *Lu*. *sherlocki*, 1) and 8 individual samples (*Pi*. *nevesi*, 1; *Ny*. *whitmanni*, 2; *Pa*. *aragaoi*, 1; *Ev*. *saulensis*, 1; *Ny*. *antunesi*, 1; *Lu*. *sherlocki*, 1; *Ev*. *infraspionsa*, 1) were amplified for the target ITS1 region (Fig. 3). The microscopic analysis revealed that five specimens of *Ev. saulensis* had flagellated forms in the hindgut, with an infection rate of 2.4%. Of the analyzed samples, 13 sequences showed similarity (99%) with *L*. (*V*.) *braziliensis* as follows: *Th*. *auraensis* (*n* = 1); *Ev*. *saulensis* (*n* = 2); *Ev*. *walkeri* (*n* = 1); *Ps*. *llanosmartinsi* (*n* = 1); *Pi*. *nevesi* (*n* = 2); *Ps*. *davisi* (*n* = 1); *Ps*. *ayrozai* (*n* = 1); *Pa*. *aragaoi* (*n* = 1); *Ny*. *antunesi* (*n* = 1); and *Ev*. *infraspinosa* (*n* = 1). Only a specimen of *Ps*. *ayrozai* exhibited similarity (94%) to the *L*. (*V*.) *guyanensis* sequence deposited in the GenBank database (MF802812–MF802824).

### Sand fly blood sources

**Fig. 4:**
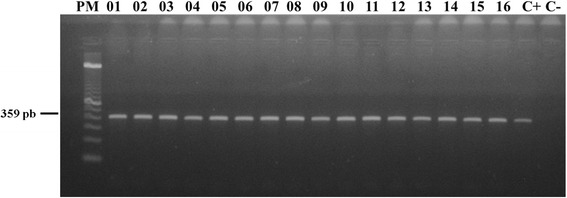
PCR-*cytb*- 2% agarose gel colored with GelRed showing *cytb* PCR amplification product from DNA extracted from female sand flies collected in the municipality of Rio Branco, Acre, Brazil. Lanes 1–16: female sand flies collected containing a blood meal; Lane C+: positive control (canine blood clot); Lane C-: negative control (Milli-Q ultrapure water)

A total of 33 fed females were submitted to amplification of the *cytb* gene, of which 25 were collected in rural areas (*Trichophoromyia* sp.: 10; *Pressatia* sp.: 2; *Ev*. *saulensis*: 7; *Ev*. *walkeri*: 1; *Ny*. *antunesi*: 1; *Ps*. *davisi*: 1; *Ps*. *hirsutus*: 1; *Ps*. *carrerai*: 1; and *Pi*. *nevesi*: 1) and eight collected in the urban park (*Trichophoromyia* sp.: 1; *Ev*. *saulensis*: 1; *Ev*. *walkeri*: 1; *Ny*. *antunesi*: 2; *Ps*. *davisi*: 1; *Pi*. *serrana*: 1; and *Mig*. *migonei*: 1 (Fig. 4). After sequencing, all samples were found to be similar to the sequence deposited in GenBank (MG027586–MG027618) for *Gallus gallus* (domestic chicken).

## Discussion

In the state of Acre, ATL and environmental impacts have historically been related to the processes of human occupation, which from the 1970’s experienced a gradual conversion from the extractive model of the region to agricultural activity [28]. These environmental changes, associated with the construction of rural settlements, urbanization and massive logging with a view to human occupation, played a major role in the dissemination of this disease in the region and the state. The impacts resulting from the dynamics of land use in the Amazon have had environmental consequences such as high rates of deforestation, loss of biodiversity, adaptation of vector species to peridomestic environments, potentially leading to extinction of rare species or increasing others.

The increase in ATL cases reported in the North Region of Brazil suggests an adaptation of sand flies to transmit ATL agents in the peridomestic environment [11], also as a consequence of the impact of anthropic activities on the environment, including animal farming near houses. This may have favored the adaptation of the vectors to human dwellings and modified the transmission cycle of ATL [36]. Studies of geographical distribution [16], ecological indices of the fauna and rates of natural infection rates are scarce in Brazil. In this study, a total of 2515 sand flies were captured and the most abundant species sampled were *Th. auraensis*, indistinguishable females of *Trichophoromyia* sp., *Ev. saulensis* and *Pressatia calcarata*, altogether representing 73.8% of the collections. Of these, *L*. (*V*.) *braziliensis* was detected in *Th. auraensis* and *Ev. saulensis*, corroborating other studies in the state that point to *Th. auraensis* as an abundant species in the sampled environments in Acre [20–22, 26] and its potential involvement in the transmission cycle of *Leishmania* spp. Recently, in the municipality of Assis Brasil, this species was found infected with *L*. (*V*.) *braziliensis* and *L*. (*V*.) *guyanensis* by PCR-RFLP [22], indicating its importance in maintaining the circulation of *Leishmania* spp., although its vectorial competence has not yet been proven [37]. In Madre de Dios, Peru, *Th. auraensis* has been found infected with *L*. (*V*.) *lainsoni* and *L*. (*V*.) *braziliensis* [38], reinforcing the importance of this species and its possible capacity as a vector in these regions of the Amazon Basin.

Among potential vectors of ATL agents to humans, *Ev. saulensis* has not been considered of medical importance. Although it has a large distribution from central Brazil to Central America, this species is not commonly collected in sand fly studies. However, this species has been found infected with *L*. (*V*.) *braziliensis* in Rio Branco [26], and in the present study was the third most frequent species in light-trap collections, the most abundant species in the collections using Shannon, being present in all environments sampled and with dominance in the forest environment, confirming its anthropophilic behavior. We also found flagellated forms in five specimens of *Ev. saulensis*, reinforcing its participation in the enzootic transmission cycle of ATL and the need for studies of the vectorial capacity and competence in transmitting *Leishmania* to humans. Furthermore, the potential participation of *Ev. saulensis* in the transmission of ATL agents, in addition to the vectors already incriminated needs to be investigated within the state of Acre. The genus *Psychodopygus* was the most dominant genus in the collections, with a total of seven species collected, of which *Ps*. *carrerai carrerai* and *Ps*. *davisi* were the most abundant in the study. Species of this genus are frequent in faunal studies of this region and have been found naturally infected in the Amazon Region [39–42]. In our studies, *Psychodopygus davisi* was found infected with *L.* (*V*.) *braziliensis*, which corroborate a previous study carried out in Assis Brasil where parasite of the *L*. (*V*.) *braziliensis* complex was found [22]. Reinforcing the importance of this species in the region is that it has been found naturally infected with *Leishmania* sp. in the state of Rondônia [43, 44], and also infected by *L*. (*V*.) *braziliensis* in the Serra dos Carajás, Pará and in the neighboring country of Peru [38, 42]. *Psychodopygus davisi* has been frequently found in peridomesitc environments, suggesting its adaptation to anthropic environments. Based on its anthropophilic behavior, density and occurrence of natural infection of *Leishmania*, this species is incriminated in the transmission cycle of ATL in the Amazon Region [37, 43–45].

The species *Ny*. *whitmani*, *Ny*. *antunesi*, *Bi*. *flaviscutellata*, *Ps*. *hirsutus hirsutus*, *Ps*. *ayrozai* and *Migonemyia migonei* were found in low abundance in the different environments studied, yet these are considered vectors of species of *Leishmania* in the region [20]. *Psychodopygus ayrozai* was found naturally infected with *L*. (*V*.) *guyanensis*, reinforcing its role in the etiological agent’s circulation of ATL in the state. In other studies, *L*. (*V*.) *guyanensis* was isolated from human cases and in sand flies already described in other epidemiological studies [18, 22]. *Psychodopygus ayrozai* is common in the North Region of Brazil [6], especially in peridomiciliar areas, indicative of its high degree of anthropophilia [14]. In the Amazon Region, species of *Psychodopygus* are associated with ATL transmission and can be important vectors in zoonotic and enzootic cycles [46]. *Thricophoromyia ubiquitalis* was described as a vector *of L*. (*V*.) *lainsoni* in the state of Pará [16, 46]. In a study carried out near the Solimões River (AM), *Th*. *ubiquitalis* was found to be the predominant species, accounting for 57.94% of flies sampled, and was also found naturally infected by *L*. (*V*.) *lainsoni* [47]. In our study, only one specimen of *Th. ubiquitalis* was captured in a Shannon trap. Faunal studies of sand flies in three municipalities of Acre identified 52 species, among them *Th. auraensis*, *Ny. antunesi*, *Ny. whitmani*, and *Ps. davisi* were responsible for 66.95% of the collected flies, with *Ny*. *whitmani* being the most abundant [20]. In the present study, these species represented 40.7% of the species collected, with the most frequent being *Th*. *auraensis* (33.3%). In 2008, Silva-Nunes et al. [16] investigating the epidemiology of ATL and surveying the phlebotomine fauna in order to identify possible vectors in the municipality of Acrelândia, collected 14 species, three of which are known vectors of the disease: *Ny. antunesi* (59.1%), in the peridomestic environment and forests edges; *Ny*. *whitmani*, more frequent in peridomestic environment (15%) and the only one within a house; and *Th. ubiquitalis*, also in peridomestic environment. In this study, *Ny*. *whitmany* and *Ny*. *antunesi* were more frequent in forest environments.

In a faunal survey of three areas of the city of Rio Branco during the course of a single year, Araújo-Pereira et al. [21] found high diversity of sand flies, with the most abundant being *Th*. *auraensis* and *Ny*. *whitmani*, representing 72% of the collected specimens. Some of the species collected by these authors were already known as vectors of the parasite that causes ATL (i.e. *Ny*. *whitmani*, *Ny*. *antunesi* and *Bi*. *flaviscutellata*). These data corroborate the high frequency of *Th. auraensis* (33.3%) and other species known to be potentially vectors of *Leishmania* parasites to humans.

In Assis Brasil, Teles et al. [22] evidenced a rich and diverse sand fly fauna of 67 species, of which *Th*. *auraensis/ruifreitasi* and *Ps*. *davisi* were dominant. These species were collected in domestic and peridomestic forest environments, demonstrating their potential as vectors of ATL agents in the region.

In the present study, species diversity was lower in the rural area than in the urban forest, with a lower index of equitability due to high species dominance. Thus, sand fly frequency seems to be related to levels of environmental degradation and food supply provided by animals in peridomestic environments, with changing the behavior of those populations compared with sand flies in unaltered environments [48]. Thus, an environmental park interconnected to an area of environmental protection, even with low frequency and lower richness, suffers less anthropic effects, and thus tends to maintain the balance on species distribution in the ecosystem. The ecological indices in the different studied ecotopes did not show any significant differences, having the greatest diversity for primary forest in the urban park. Silva et al. [48] suggested that areas with vegetation in different successional stages may influence the dominance and frequency of sand flies. Also, high species diversity is common in the Amazon Region, as observed by other authors [37, 43, 47].

There was an increase in the abundance of sand flies collected with light traps at the beginning of the rainy season, from December to April, with smaller quantities during the dry months with low humidity and the occurrences of Amazonian cool periods. The species frequency had a significant positive correlation with temperature variation (Spearman’s rho = 0.58, *P* = 0.030), indicating that with increasing temperature there is an increase for abundance of collected sand flies. Other studies have recognized a similar pattern of seasonal variation in sand flies such in Campo Grande, Mato Gross do Sul, with an increase in the abundance of captured specimens after the months of highest rainfall and humidity [49]. It should be noted that there are distinct seasonal patterns for sand flies in different regions of Brazil. In Rio Grande do Norte, a study found that sand flies were most abundant during the dry season, and declined in abundance in the rainy season when temperatures decreased and relative humidity was high [50].

All fed female sand flies submitted to analysis of blood sources exhibited an amplified gene region compatible with *Gallus gallus* (domestic chicken). Although chickens do not act as reservoirs of *Leishmania* spp., their presence may influence on the population dynamics of sand flies with increasing population density in the peridomestic environment, which could contribute to increase the risk of the transmission of *Leishmania* to humans [51]. The presence of animal shelters (e.g. stables and chicken pen) in the peridomestic environment represents a permanent food supply and provides shelter for sand flies, as observed in studies in the State of Maranhão, where sand fly sampling was unsuccessful in environments without the presence of domestic animals [52]. Again, the presence of large numbers of chickens in peridomestic environments can be highly attractive to sand flies and may contribute to the maintenance of their life cycle, and thus constitute an epidemiological problem by keeping the vector near the human environment [52, 53]. It is worth noting that the presence of infected sand flies and the presence of synanthropic reservoirs can increment the transmission cycle in the peridomestic environment of the present study. Thus, in this study, domestic chicken appears to be a common blood source for sand flies, probably because it does not offer resistance and are kept in large number in the study area [53].

## Conclusions

The sand fly fauna found in the present study was composed of 43 species and included known vectors of ATL, such as *Nyssomyia whitmani*, *Ny*. *antunesi*, *Bichromomyia flaviscutellata* and *Mg*. *migonei*. The high frequency of *Trichophoromyia auraensis* and *Evandromyia saulensis*, and the detection of *L*. (*V*.) *braziliensis* DNA, and *Ps. ayrozai* with *L*. (*V*.) *guyanensis* DNA*,* indicate that these species could be putative vectors for ATL in this Amazonian region. Investigation of blood sources of sand flies revealed a preference among female sand flies collected in this area for domestic chicken, which may be participating in the population dynamics of these insects.

## References

[1] Brasil. Ministério da Saúde. Secretaria de Vigilância em Saúde.Guia de Vigilância em Saúde / Ministério da Saúde, Secretaria de Vigilância em Saúde. – Brasília: Ministério da Saúde; 2014. p. 812

[2] Lessa MM, Lessa HA, Castro TWN, Oliveira A, Scherifer A, Machado P (2007). Leishmaniose mucosa: aspectos clínicos e epidemiológicos. Rev Bras Otorrinolaringol.

[3] Gontijo B, Carvalho MDL (2003). Leishmaniose tegumentar americana. Rev Soc Bras Med Trop.

[4] Lainson R, Shaw JJ. New world Leishmaniasis. In: Cox FEG, Kreier JP, Wakelin D, editors. Topley & Wilson’s Microbiology and Microbial Infections. Wiley; 2005. p. 313–49.

[5] Young DG, Duncan MA (1994). Guide to the identification and geographic distribution of *Lutzomyia* sand flies in Mexico, West Indies, central and South America. Mem Am Entomol Inst.

[6] Brazil RP, Rodrigues AAF, Filho JDA. Sand fly vectors of *Leishmania* in the Americas - a mini review. Entomol Ornithol Herpetol. 2015;4:144.

[7] Brasil. Ministério da Saúde. Sistema de Informação de Agravos de Notificação - SINAN. http://portalsinan.saude.gov.br/. Accessed 28 Nov 2016.

[8] Patz JA, Graczyk TK, Geller N, Vittor YA (2000). Effects of environmental changes on emerging parasitic disease. Int J Parasitol.

[9] Petney TN (2001). Environmental, cultural and social changes and their influence on parasite infections. Int J Parasitol.

[10] Molyneux DH (2006). Control of human parasitic diseases: context and overview. Adv Parasitol.

[11] Silva NS, Muniz VD (2009). Epidemiologia da leishmaniose tegumentar americana no Estado do Acre, Amazônia brasileira. Cad de Saúde Pública.

[12] Ximenes MFFM, Silva VPM, Queiroz PVS, Rego MM, Cortez AM, Batista LMM (2007). Flebotomíneos (Diptera: Psychodidae) e leishmanioses no Rio Grande do Norte, Nordeste do Brasil – reflexos do ambiente antrópico. Neotrop Entomol.

[13] Ambroise-Thomas P (2000). Emerging parasite zoonoses: the role of host-parasite relationship. Int J Parasitol..

[14] Basano SA, Camargo LMA (2004). American cutaneous leishmaniasis: history, epidemiology and prospects for control. Rev Bras Epidemio.

[15] Silva NS, Viana AB, Cordeiro JA, Cavasini CE (1999). Leishmaniose tegumentar americana no estado do Acre. Brasil. Rev Saúde Públ..

[16] Silva-Nunes M, Cavasini CE, Silva NS, Galati EAB (2008). Epidemiologia da leishmaniose tegumentar e descrição das populações de flebotomíneos no município de Acrelândia, Acre. Brasil. Rev Bras de Epidemiol..

[17] Tojal AC, Romero GAS, Cupolillo EA (2003). A diversidade das espécies causadoras de leishmaniose cutânea em Rio Branco - Acre. Rev Soc Bras Med Trop.

[18] Tojal AC, Cupolillo E, Volpini AC, Almeida R, Romero GAS (2006). Species diversity causing human cutaneous leishmaniasis in Rio Branco, state of acre, Brazil. Tropical Med Int Health.

[19] Oliveira JGS, Novais FO, Oliveira CI, Cruz-Júnior AC, Campos LF, Rocha AV (2005). Polymerase chain reacton (PCR) is highly sensitive for diagnosis of mucosal leishmaniasis. Acta Trop.

[20] Azevedo ACR, Costa SM, Pinto MCG, Souza JL, Cruz HC, Vidal J (2008). Studies on the sandfly fauna (Diptera: Psychodidae: Phlebotominae) from transmission areas of American cutaneous leishmaniasis in state of Acre. Brazil. Mem Inst Oswaldo Cruz..

[21] Araújo-Pereira T, Fuzari AA, Andrade-Filho JD, Pita-Pereira D, Britto C, Brazil RP (2014). Sand fly fauna (Diptera: Psychodidade: Phlebotominae) in an area of leishmaniasis transmission in the municipality of Rio Branco, sate of Acre. Brazil. Parasit Vectors..

[22] Teles CBG, Santos APA, Freitas RA, Oliveira AFJ, Ogawa GM, Rodrigues MS (2016). Phlebotomine sandfly (Diptera: Psychodidae) diversity and their Leishmania DNA in a hot spot of American cutaneous leishmaniasis human cases along the Brazilian border with Peru and Bolivia. Mem Inst Oswaldo Cruz..

[23] Pessoa FAC, Medeiros JF, Barrett TV (2007). Effects of timber harvest on phlebotomine sand flies (Diptera: Psychodidae) in a production forest: abundance of species on tree trunks and prevalence of trypanosomatids. Mem Inst Oswaldo Cruz.

[24] Pinheiro FG, Luz SLB, Franco AMR (2008). Infecção natural por tripanosomatídeos (Kinetoplastida: Trypanosomatidae) em *Lutzomyia umbratilis* (Diptera: Psychodidae) em áreas de leishmaniose tegumentar americana no Amazonas, Brasil. Acta Amaz.

[25] Reis SR, Gomes LHM, Ferreira NM, Nery LR, Pinheiro FG, Figueira LP (2013). Ocorrência de flebotomíneos (Diptera: Psychodidae: Phlebotominae) no ambiente peridomiciliar em área de foco de transmissão de leishmaniose tegumentar no município de Manaus, Amazonas. Acta Amaz.

[26] Araujo-Pereira T, Pita-Pereira D, Boité MC, Melo M, Costa-Rego TA, Fuzari AA, et al. First description of *Laishmania* (*Viannia*) infection in *Evandromyia saulensi*s, *Pressatia* sp. and *Trichophoromyia auraensis* (Psychodidae: Phlebotominae) in a transmission area of cutaneous leishmaniases in Acre state, Amazon Basin, Brazil. Mem Inst Oswaldo Cruz. 2017;112:75–8. 10.1590/0074-02760160283 PMC5225531 28076470

[27] IBGE. Instituto Nacional Brasileiro de Geografia e Estatística (2016). Anuário Estatístico do Brasil.

[28] Acre (2010). Programa Estadual de Zoneamento Ecológico Econômico Fase II: documento Síntese Rio Branco.

[29] Foranttini OP (1983). Entomologia Médica IV. Psychodidae: Phlebotominae, Leishmaniose e Bartonelose.

[30] Galati EAB (2003). Morfologia e Taxonomia: Morfologia, terminologia de adultos e identificação dos táxons da América.

[31] Marcondes CB (2007). A proposal of generic and subgeneric abbreviations of phlebotomines sandflies (Diptera: Psychodidae: Phlebotominae) of the world. Entomol News.

[32] Schonian G, Nasereddin A, Dinse N, Schweynoch C, Schallig HD, Presber W, Jaffe CL (2003). PCR diagnosis and characterization of *Leishmania* in local and imported clinical samples. Diagn Microbiol Infect Dis.

[33] Steuber S, Abdel-Rady A, Clausen P-H (2005). PCR-RFLP analysis: a promising technique for host species identification of blood meals from tsetse flies (Diptera: Glossinidae). Parasitol Res.

[34] Magurran AE (2004). Measuring biological diversity.

[35] Paiva BR, Secundino NFC, Pimenta PFP, Galati EAB, Andrade-Junior HF, Malafronte RDS (2007). Standardization of conditions for PCR detection of *Leishmania* spp. DNA in sandflies (Diptera, Psychodidae). Cad Saude Pública.

[36] Costa SM, Cechinel M, Banderia V, Zannuncio JC, Lainson R, Rangel EF. *Lutzomyia* (*Nyssomyia*) *whitmani* s.l. (Antunes & Coutinho, 1939) (Diptera: Psychodidae: Phlebotominae): geographical distribution and the epidemiology of American cutaneous leishmaniasis in Brazil mini-review. Mem Inst Oswaldo Cruz. 2007;102:149–53. 10.1590/s0074-02762007005000016 17426877

[37] Ogawa GM, Pereira Júnior AM, Resadore F, Ferreira RGM, Medeiros JF, Camargo LMA (2016). Sandfly fauna (Diptera: Psychodidae) from caves in the state of Rondônia, Brazil. Braz J Vet Parasitol.

[38] Valdivia HO, De Los Santos MB, Fernandez R, Baldeviano GC, Zorrilla VO, Vera H (2012). Natural *Leishmania* infection of *Lutzomyia* (*Trichophoromyia*) *auraensis* in Madre de Dios, Peru, detected by a fluorescence resonance energy transfer-based real-time polymerase chain reaction. Am J Trop Med Hyg..

[39] Silva DF, Freitas RA, Franco AM (2007). Diversidade e abundância de flebotomíneos do gênero *Lutzomyia* (Diptera: Psychodidae) em áreas de mata do nordeste de Manacapuru, AM. Neotrop Entomol..

[40] Neto GJL, Baima JM, Freitas RAD, Passos MAB (2012). Fauna flebotomínica (Diptera: Psychodidae) em floresta preservada e alterada do Município de Caroebe, Estado de Roraima, Brasil. Rev Pan-Amaz Saude..

[41] Neto GJL, Freitas RAD, Baima JM, Passos MAB (2010). Fauna flebotomínica (Diptera: Psychodidae) da Serra do Tepequém, Município de Amajarí, Estado de Roraima, Brasil. Rev Pan-Amaz Saude..

[42] Souza AAA, Silveira FT, Lainson R, Barata IDR, Silva MDGS, Lima JAN (2010). Fauna flebotomínica da Serra dos Carajás, Estado do Pará, Brasil, e sua possível implicação na transmissão da leishmaniose tegumentar americana. Rev Pan-Amaz Saude.

[43] Gil LHS, Basano SA, Souza AA, Silva MGS, Barata I, Ishikawa EA (2003). Recent observations on the sand fly (Diptera: Psychodidae) fauna of the state of Rondônia, western Amazônia, Brazil: the importance of *Psychdopygus davisi* as a vector of zoonotic cutaneous leishmaniasis. Mem Inst Oswaldo Cruz.

[44] Grimaldi G, Momen H, Naiff RD, McMahon-Pratt D, Barrett TV (1991). Characterization and classification of leishmanial parasites from humans, wild mammals, and sand flies in the Amazon region of Brazil. Am J Trop Med Hyg.

[45] Alves VR, Freitas RA, Santos FL, Oliveira AFJ, Barrett TV, Shimabukuro PHF (2012). Sand flies (Diptera, Psychodidae, Phlebotominae) from Central Amazonia and four new records for the state of Amazonas, Brazil. Rev Bras Entomol.

[46] Silveira FT, Souza AAA, Lainson R, Shaw JJ, Braga RR, Ishikawa EA (1991). Cutaneous leishmaniasis in the Amazon region: natural infection of the sandfly *Lutzomyia ubiquitalis* (Psychodidae: Phlebotominae) by *Leishmania* (*Viannia*) *lainsoni* in Pará state, Brazil. Mem Inst Oswaldo Cruz.

[47] Pereira AM, Teles CBG, dos Santos APA, Souza Rodrigues MD, Marialva EF, Pessoa FAC (2015). Ecological aspects and molecular detection of *Leishmania* DNA Ross (Kinetoplastida: Trypanosomatidae) in phlebotomine sandflies (Diptera: Psychodidae) in terra firme and várzea environments in the middle Solimões region, Amazonas state, Brazil. Parasit Vectors.

[48] Silva AMD, Camargo NJD, Santos DRD, Massafera R, Ferreira AC, Postai C (2008). Diversidade, distribuição e abundância de flebotomíneos (Diptera: Psychodidae) no Paraná. Neotrop Entomol.

[49] Oliveira AGD, Andrade Filho JD, Falcão AL, Brazil RP (2003). Estudo de flebotomíneos (Diptera, Psychodidae, Phlebotominae) na zona urbana da cidade de Campo Grande, Mato Grosso do Sul, Brasil, 1999-2000. Cad Saúde Pública..

[50] Pinheiro MPG, Silva JHT, Cavalcanti KB, de Azevedo PRM (2013). Ecological interactions among phlebotomines (Diptera: Psychodidae) in an agroforestry environment of Northeast Brazil. J Vector Ecol.

[51] Teodoro U, La Salvia FV, Lima EM, Spinosa RP, Barbosa OC, Ferreira MEMC (1993). Observações sobre o comportamento de flebotomíneos em ecótopos florestais e extraflorestais, em área endêmica de leishmaniose tegumentar americana, no norte do Estado do Paraná, sul do Brasil. Rev Saúde Pública.

[52] Dias FDOP, Lorosa ES, Rebêlo JMM (2003). Fonte alimentar sangüínea e a peridomiciliação de *Lutzomyia longipalpis* (Lutz & Neiva, 1912) (Psychodidae, Phlebotominae). Cad Saúde Pública.

[53] Brazil RP, De Almeida DC, Brazil BG, Mamede SM (1991). Chicken house as a resting site of sandflies in Rio de Janeiro, Brazil. Parassitologia.

